# Impact of climate change on grape berry ripening: An assessment of adaptation strategies for the Australian vineyard

**DOI:** 10.3389/fpls.2022.1094633

**Published:** 2022-12-21

**Authors:** Suzy Y. Rogiers, Dennis H. Greer, Yin Liu, Tintu Baby, Zeyu Xiao

**Affiliations:** ^1^ New South Wales Department of Primary Industries, Wollongbar, NSW, Australia; ^2^ Australian Research Council Training Centre for Innovative Wine Production, Urrbrae, SA, Australia; ^3^ Gulbali Institute, Charles Sturt University, Wagga Wagga, NSW, Australia; ^4^ School of Agriculture Environmental and Veterinary Science, Charles Sturt University, Wagga Wagga, NSW, Australia

**Keywords:** viticulture, management systems, adaptation, drought, heat stress, irrigation, soil health, *Vitis vinifera*

## Abstract

Compressed vintages, high alcohol and low wine acidity are but a few repercussions of climate change effects on Australian viticulture. While warm and cool growing regions may have different practical concerns related to climate change, they both experience altered berry and must composition and potentially reduced desirable wine characteristics and market value. Storms, drought and uncertain water supplies combined with excessive heat not only depress vine productivity through altered physiology but can have direct consequences on the fruit. Sunburn, shrivelling and altered sugar-flavour-aroma balance are becoming more prevalent while bushfires can result in smoke taint. Moreover, distorted pest and disease cycles and changes in pathogen geographical distribution have altered biotic stress dynamics that require novel management strategies. A multipronged approach to address these challenges may include alternative cultivars and rootstocks or changing geographic location. In addition, modifying and incorporating novel irrigation regimes, vine architecture and canopy manipulation, vineyard floor management, soil amendments and foliar products such as antitranspirants and other film-forming barriers are potential levers that can be used to manage the effects of climate change. The adoption of technology into the vineyard including weather, plant and soil sensors are giving viticulturists extra tools to make quick decisions, while satellite and airborne remote sensing allow the adoption of precision farming. A coherent and comprehensive approach to climate risk management, with consideration of the environment, ensures that optimum production and exceptional fruit quality is maintained. We review the preliminary findings and feasibility of these new strategies in the Australian context.

## 1 Emerging challenges

Terroir is the result of an interaction between climate, soil, landscape characteristics, topography and biodiversity for a particular cultivar within the vineyard and aside from the inherent natural environment, it also encompasses the cultural management of a site. It refers to “the interactions between the identifiable physical and biological environment and applied vitivinicultural practices, providing distinctive characteristics for the products originating from this area” (OIV, 2010). Of these factors, temperature is undoubtedly a strong driving force for vine and fruit development (Jones and Davis, 2000; Jones and Alves, 2012). The Mediterranean climate is considered ideal for viticulture. Hence, warm, dry summers are accompanied by cool, wet winters and these combinations of temperature, light and water drive the desirable evolution of berry aroma, colour and flavour in hundreds of grape cultivars (Keller, 2010). That said, grapevines are grown with economic success across a range of climatic zones, resulting in highly diverse wine styles (Van Leeuwen et al., 2004). However, heat, drought, wildfires, excessive rain events and increased pest and disease pressure are posing new challenges for viticulture. Additionally, many viticultural regions are consistently experiencing a general phenological advancement in flowering, veraison and maturity. These trends and emerging challenges have been, at least partially, attributed to a changing climate (Caffarra and Eccel, 2010; Bonnefoy et al., 2013; Malheiro et al., 2013; Cola et al., 2017; Jarvis et al., 2017; Alikadic et al., 2019; Cameron et al., 2022).

Alongside the ambient temperature rise, Australia has been subjected to more extreme climatic events like heatwaves, wildfires and shifts in the timing and volume of rain (Abram et al., 2021). In February 2009, Eastern Australia witnessed extreme temperatures and one of the most devastating fires on record. Another extreme heat event occurred in December 2012-January 2013 across 70% of Australia with temperature records in every state and territory. The maximum temperature averaged across Australia was the hottest ever recorded at 40.3°C. Furthermore, a frost in November 2017 across South Australia wiped out 30,000 tonnes of grapes, while hail during flowering in 2017 in the Riverina region of NSW resulted in complete crop loss. These are genuine current examples of extreme climatic effects on viticultural productivity. Additionally, the unprecedented 2019-20 Black Summer bushfires occurred during record breaking temperatures and very low rainfall. These fires were classified as ‘megafires’ with nearly 19 million hectares of land destroyed and with an extreme impact on biodiversity and at least one billion vertebrate animals lost (Filkov et al., 2020). This disaster also resulted in extensive social and economic impacts, including smoke-related effects on public health and on vineyards in many of the affected wine-growing regions.

Australian viticulture stretches from the southernmost latitudes of Victoria, South Australia and Western Australia northward into New South Wales and Queensland and it has been projected that by 2050 a warming climate will reduce the suitable area available for cultivation by 22-73% (Hannah et al., 2013); this is despite factoring in estimates of emerging novel areas for cultivation. These projections, however, are based on existing cultivars and current management strategies. The adoption of new cultivars and integration of adaptive measures to tackle these mounting climate pressures will certainly lessen the severity of these predictions (Van Leeuwen et al., 2013; Mosedale et al., 2016). However, research and knowledge transfer are required now allowing appropriate practical strategies to be implemented (Santos et al., 2020).

### 1.1 Heat stress

The growing season mean temperature is an important driver for root, canopy and reproductive development. Grapevines can be cultivated in average growing season temperatures with a lower and upper threshold of 12-13°C and 22-24°C (Schultz and Jones, 2010). Once endo- and eco-dormancy are broken, temperatures above 7-10°C will drive budburst (Amerine and Winkler, 1944), followed by new vegetative growth and the emergence of the inflorescences. Warm temperatures will encourage development of the canopy, but later in the season, if temperatures reach above 35°C, heat stress will impact on the physiology of the vine. Temperatures can exceed 40°C for prolonged periods in Australia and this will have an impact on carbon assimilation and thus sugar accumulation by grapes (Greer and Weston, 2010). The process of photosynthesis is vulnerable to temperatures due reductions in carboxylation by ribulose 1,5-bisphosphate carboxylase/oxygenase (Rubisco) and the regeneration of ribulose 1,5-bisphosphate (RuBP) (Greer and Weedon, 2012). Heat is also often combined with low humidity and therefore, to prevent water stress, stomata will close.

The berries themselves are vulnerable to heat stress with repercussions on berry composition and wine quality. Aside from impact on primary metabolites such as sugars (Greer and Weston, 2010; Pillet et al., 2012), organic acids (Sweetman et al., 2014) and amino acids (Lecourieux et al., 2017), secondary metabolites responsible for the sensory attributes are also altered. Flavonoids are affected by high temperatures, but outcomes depend on the heat intensity, duration, phenological stage and genotype (Gouot et al., 2019). Anthocyanin changes were attributed to a combination of changes in gene expression, enzyme activity, degradation and relocation (Gouot et al., 2019). Additionally, molecular data pointed toward cell wall changes (Lecourieux et al., 2017) with potential ramifications on berry texture. Cell walls of berry skin cells became more extensible perhaps to enable berry contraction and expansion to occur between the day and night extremes. Elevated canopy temperature can accelerate late ripening mesocarp cell death and berry desiccation (i.e. shrivelling) in susceptible cultivars (Bonada et al., 2013a). High temperature may exacerbate late ripening cell death by increasing respiration within the berries. Due to reduced oxygen diffusivity in late ripening berries, the mesocarp can become hypoxic, resulting in localised anaerobic fermentation which leads to ethanol accumulation (Xiao et al., 2018b). Excessive radiation, in combination with heat, can result in sunburn, especially on the western side of the canopy (Greer and Weedon, 2013; Greer et al., 2006). Sun exposure and the microclimate of the bunch will dictate berry temperature and, therefore, careful manipulation of the canopy may play an important role in berry attributes, and indeed berry survival during an extreme temperature event.

### 1.2 Water deficits

Water deficits can impact on vegetative growth, inflorescence development, berry set and berry development, though dependent on the phenological stage, severity and duration of the water deficit (Hardie and Considine, 1976). Photosynthesis occurs at the expense of transpiration, with the efficiency of carbon gain dependent on cultivar. Mild deficits may result in stomatal limitations, but more severe deficits will result in non-stomatal limitations, affecting photosynthesis (Lovisolo et al., 2010). Drought tolerant cultivars with adequate stomatal control and water-use efficiency are a priority; as leaf water potential declines, hydraulic conductivity can by maintained through stomatal closure. Shiraz and Semillon, for instance, are situated on the anisohydric end of the isohdyric to anisohydric spectrum and can suffer from water stress (Schultz, 2003; Rogiers et al., 2009). However, cultivar behaviour is not always clearly aligned to one or the other end of the spectrum, and may be inconsistent (Charrier et al., 2018). Stomatal regulation and transpiration are strongly influenced by the environment, even during the night (Rogiers and Clarke, 2013). However, unfavourable conditions may not necessarily increase the risk of plant water stress (Dayer et al., 2021). For instance, the conditions preceding the water stress and the rate at which the water stress is imposed will influence the outcomes (Morabito et al., 2022). The age of the plant and the phenological stage also play a role. Water stress will hamper cell division and elongation, and thus overall growth and reproductive development. To cope with the increase in osmotic stress as a result of dehydration, cells can accumulate osmoprotectants such as sugars and amino acids. Leaf petiole ABA (abscisic acid) concentrations are positively correlated with root sucrose concentrations in water stressed Grenache and Semillon, indicative of integration between ABA signalling and carbohydrate metabolism (Rogiers et al., 2011a) during water stress conditions. Water stress can also accelerate late ripening mesocarp cell death and exacerbate the berry dehydration effect in prone cultivars (Fuentes et al., 2010; Bonada et al., 2013b), resulting in increased sugar concentration (Sadras and McCarthy, 2007; Caravia et al., 2016), altered chemical composition (Šuklje et al., 2016) and sensory characteristics (Bonada et al., 2013b) of the berry.

### 1.3 Bushfires

The risk of fire is predicted to intensify in Australia as a result of rising temperature (Williams et al., 2001; Clarke et al., 2013; Abram et al., 2021). The fire season will likely increase in both duration and intensity resulting in bushfires that damage vines directly or compromise the crop through smoke exposure (Summerson et al., 2021). Smoke exposed fruit resulted in undesirable wine aromas with their intensity dependent on the phenological stage, characteristics of the fire and environmental conditions (Kennison et al., 2007; Krstic et al., 2015). The volatile phenols guaiacol, 4-methylguaiacol, *o*-, *m*-, and *p*-cresol; and syringol specifically contributed to the smoky aroma (Jones et al., 2022). These volatile phenols enter the fruit through their cuticle, with little transport from leaves. Once inside the berry, the phenols are glycosylated as a detoxifying mechanism (Dungey et al., 2011; Noestheden et al., 2018; Jones et al., 2022). Both the free and glycosylated forms have repercussions on aroma and flavour (Mayr et al., 2014).

### 1.4 Waterlogging

Heavy rainfall events and flooding are becoming more frequent and more intense globally, including Australia (Hague, 2021). Flooding as a result of excessive rain may lead to plant oxidative stress as a result of hypoxia and/or anoxia. The transport of O_2_ from the leaves to the roots becomes insufficient because O_2_ is consumed enroute, and there is a large resistance to gas movement in water saturated root conditions. Moreover, soil microorganisms compete with the roots for any remaining oxygen (Sauter, 2013). Once hypoxic conditions are perceived, a cascade of events led by hypoxic genes are switched on in all the plant’s organs (Ruperti et al., 2019). Reduced oxygen levels in the roots results in lowered ability for aerobic respiration, however, alcoholic fermentation is able to generate limited energy. Toxic metabolites and reactive oxygen species (ROS) can accumulate under hypoxia or anoxia (Ponnamperuma, 1972). Moreover, the translocation of carbohydrates from the reserve sites in the roots to the rest of the plant may be hampered or rapidly utilised *in situ* (Sauter, 2013). Reduced hydraulic conductance as a result of insufficient aquaporin activity (Colmer and Greenway, 2011) may further result in stomatal closure and consequently wilting. Canopy senescence, decreased root growth and root decay may also ensue. However, under adequate carbon supplies, new adventitious roots can be produced to maintain oxygen delivery (Striegler et al., 1993). In general, roots under hypoxic conditions are less able to take up water and macronutrients (Bailey-Serres and Voesenek, 2010). Soil microorganisms also require oxygen and nitrate availability declines as a result of less microbiological nitrification (Nguyen et al., 2018). The combined carbohydrate and nutrient starvation may eventually result in the death of the vine following flooding-induced hypoxia and anoxia.

### 1.5 Oxidative stress

Biotic and abiotic stress such as heat, drought, waterlogging, UV-B radiation, nutrient imbalances and salinity can result in the overproduction of ROS, leading to oxidative stress. ROS include hydrogen peroxide (H_2_O_2_), superoxide, singlet oxygen, the hydroxyl radical and organic and inorganic peroxides. ROS propagate chain reactions to target nucleic acids, lipids, proteins and other biomolecules causing oxidative damage (Sharma et al., 2012). However, depending on the concentration, ROS are also secondary messengers for cellular processes, including stress responses, and it is the delicate equilibrium between scavenging and production that determine their role (Mittler et al., 2022). Baseline levels of ROS are produced in most cell compartments, including the chloroplast and the mitochondrion in the processes of photosynthesis and respiration, and under normal conditions cellular homeostasis is maintained. Elevated cellular oxidation plays multiple roles in grapevine growth and development. Bud burst was suggested to be associated with a localised modulation of oxidative signalling within the developing cambium and vascular tissues of the enclosed meristem (Meitha et al., 2015). The onset of berry ripening is linked to the accumulation of H_2_O_2_ in the skin, in Pinot Noir (Pilati et al., 2014). Under stress conditions, ROS production and accumulation is further enhanced. For instance, under excessive heat, ROS accrue in the cytosol and the nucleus (Babbar et al., 2021). As a result of stomatal closure in response to drought, excess radiative energy can cause oxidation and hence impair the chloroplast, apoplast and cytosol. Because plants are often exposed to several stresses simultaneously (e.g. drought, high light and heat), overproduction of ROS may result in cell death and tissue necrosis. Cellular oxygen sensing (Xiao et al., 2018a; Xiao et al., 2018b), ROS detection technology (Chen and Fluhr, 2018) and molecular tools, including DNA, RNA and proteins, can be implemented to better understand vine response to heat and drought and conditions resulting in oxidative stress (Gomès et al., 2021).

### 1.6 Earlier maturity and decoupling of phenolic from sugar ripeness

Earlier grape maturity occurring than in the recent past is manifesting in many viticulture regions (Duchêne and Schneider, 2005; Wolfe et al., 2005; Etien et al., 2009; Duchêne et al., 2010; Urhausen et al., 2011; Webb et al., 2011; Morales-Castilla et al., 2020). For example, studies found that fruit maturity has advanced by 8 days per decade between 1985 and 2009 in southern Australia (Webb et al., 2011; Webb et al., 2012), while another estimated advancement by 0.5 to 3.1 days per year over 1993 to 2006 (Petrie and Sadras, 2008). In Chardonnay, Shiraz and Cabernet Sauvignon of south-eastern Australia, the early maturity is driven by the early onset of ripening as opposed to faster ripening (Sadras and Petrie, 2011). Earlier ripening may be driven by temperature, but also by vine water stress as dry soils can stimulate the production of the ripening hormone ABA. Optimum sugar levels in these warmer seasons are, however, not always concomitant with similar maturity in colour, flavour or aroma (Kliewer and Torres, 1972; Sadras and Moran, 2012; Sadras et al., 2013), culminating in the suggestion that ‘sugar ripeness’ is no longer co-ordinated with ‘phenolic’ ripeness (Van Leeuwen and Seguin, 2006; Goode, 2012). In other words, the anthocyanins and tannins have not matured to the same extent as they would in cooler years characterised by slower sugar accumulation. Berry acidity is another important quality parameter likely to decline in response to warming (Leolini et al., 2019). Warm nights can result in the respiratory loss of malic acid with the effect that the sugar-acid and aroma-acid balance is no longer optimal (Gatti et al., 2015). However, it has been suggested the trends in earlier ripening may not solely be the consequence of climate change, considering that better disease management, fertilizer application and deliberate yield reductions (to achieve an appropriate leaf area to yield ratio) have been implemented over the last decades (Webb et al., 2012). Regardless, the shift in phenology as a result of climate change demands viticultural practices to counteract these negative effects on vine resilience and berry attributes. A study of Australian premium wine found that quality ratings captured the impact of weather on wine prices (Oczkowski, 2016). It was also noted that production occurs at seasonal temperatures that are warmer than optimal. This is likely to be exacerbated with climate warming and will have economic repercussions.

### 1.7 Compressed vintages

A compressed vintage refers to a shorter harvest window for one particular cultivar. It also refers to a narrower harvest window for several cultivars in one particular region (Sadras et al., 2013; Petrie and Sadras, 2016), bringing about competition for labour, harvesting machinery, cooling capacity, winery processing equipment and tank space (Sadras et al., 2013). The consequence is that harvest compression may result in extended ‘hang time’ due to the inability of growers and wineries to process large amounts of fruit at once. This extended ripening period can lead to overripe fruit with berry shrivelling (desiccation), the concentration of existing sugars so that alcohol levels rise, loss of acidity and even the degradation of anthocyanins as well as altered flavour and aroma profiles such as a loss of fresh fruit characters (Bonada et al., 2013a; Bonada et al., 2013b; Šuklje et al., 2016). Moreover, alcohol levels have been rising steadily across many wine regions (Alston et al., 2011). In Languedoc, France alcohol increased from 11 to 14%, pH increased from 3.5 to 3.75 and total acidity dropped from 6.0 to 4.5 g/L (Van Leeuwen et al., 2019). Bordeaux wines typically were 12.5% in the 1980s but today have risen to 16%. In Australia, alcohol concentrations for red wines have increased from 12.4% in 1984 to a peak of 14.5% in 2005 (Godden and Muhlack, 2010). These high alcohol levels are considered less ‘food-friendly’ (Jones, 2010; Jones, 2012), and considering the societal issues associated with high alcohol and growing trend for healthier lifestyles, consumers are expressing an interest in lower alcohol levels (Saliba et al., 2013).

### 1.8 Pest and disease pressure

Shifts in phenology and the geographic distribution of grapevine pest insects are taking place as a result of direct and indirect effects of climate change (Salinari et al., 2006; Reineke and Thiéry, 2016). The spatial and temporal distribution of insects and pathogens is largely determined by temperature, light and water considering these factors control their growth and development (Rosenzweig et al., 2001). These factors also modify the physiology and resilience of the grapevine as the host. Drought exacerbates underlying issues such as trunk diseases (Eutypa and Botryospheria), nematodes and borers while wet conditions are conducive to increased disease activity such as powdery and downy mildew, Botrytis and other bunch rots (Salinari et al., 2006; Steel et al., 2011; Galarneau et al., 2019). Drier conditions and reduced leaf wetness may lead to decreased infections; however, the accompanying warmer springtime temperatures will negate this due to earlier infections (Salinari et al., 2006). Because grapevines may become vulnerable to new pathogens as they spread geographically, regular monitoring and an adaptive environmentally conscious preventative program will be essential.

### 1.9 Changing soil properties and dynamics

The physicochemical and biological properties of the soil have a strong influence on soil quality and functionality, and these impact on vine physiology and therefore yield and grape quality (Cataldo et al., 2021). Soil is a living biological entity with multiple functions beyond crop productivity (Doran, 2002; Lal, 2016). The soil contributes to water, carbon and mineral cycles, maintains essential ecosystem functionality by maintaining air and water quality, and providing a habitat for biodiversity (Yang et al., 2020). Analogous to rising air temperatures, the temperature of the soil has increased over the past decades in certain regions (Zhang et al., 2005), and high air temperatures have been associated with record soil temperatures (Ooi et al., 2009). To what extent cover crops ameliorate the relationship between air and soil temperature need investigation. However, soil temperature is an important driver for microbial activity (Bradford et al., 2019), carbon sequestration/release (Huang et al., 2018), greenhouse gas emissions (Smith et al., 2018), and below- and above-ground growth (Rogiers et al., 2014). Warmer soils can initiate earlier budbreak (Rogiers et al., 2014) and this may increase susceptibility of vine shoot growth to late-spring frosts.

The increasing variability in the quantity, intensity of rain and other extreme weather events also influence soil health (Allen et al., 2011). The physical loss of soil through mechanical cultivation and displacement through erosion is likely to be exacerbated by heavy rains. Prolonged drought will impact on water infiltration, soil organic matter and carbon sequestration. Additionally, low soil moisture decreases N availability in the upper soil horizons (Curtin et al., 2012). N is important to overall growth and berry composition such as aromatic secondary metabolites as well as yeast assimilable nitrogen (YAN) for fermentation. Drought will also influence the species and quantity of functional micro-organisms present because water acts as a resource, solvent and transport medium (Schimel, 2018). Aside from the soil itself, the composition and quantity of root exudates are affected by environmental parameters such as temperature and water, and these interact with the root microbiome to influence nutrient cycling, organic matter decomposition and plant growth regulation (Yang et al., 2020).

## 2 Intervention strategies

**Figure 1:**
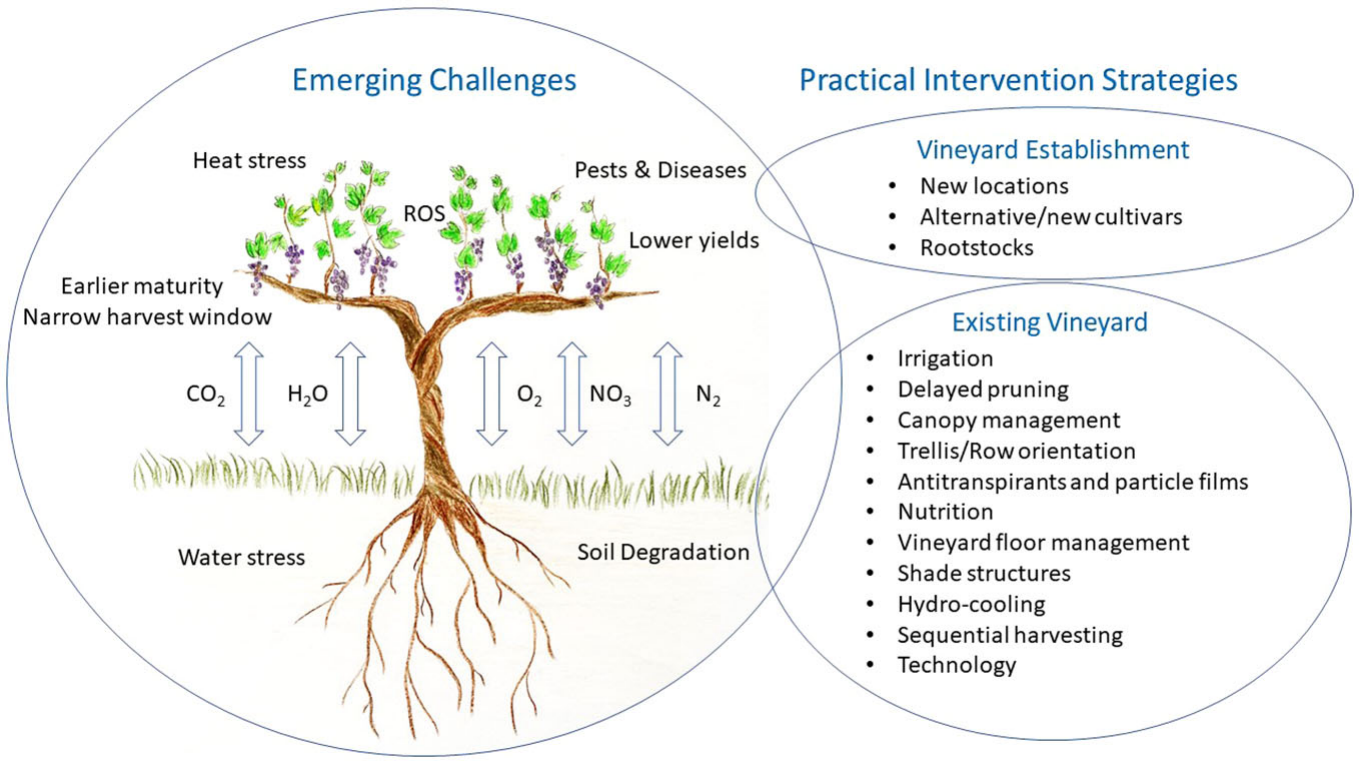
Summary of emerging challenges related to climate change and potential practical intervention strategies for the viticulture industry to consider.

Without intervention, the combination of these stresses may lead to greater yield variability, sub-optimal berry composition and inconsistency in wine style and typicity. Aside from accessing cooler regions, these approaches could include: alternative cultivars and rootstocks, efficient irrigation strategies, delayed pruning, and consideration of row orientation, training systems along with canopy manipulation, plant tissue films, sustainable vineyard floor management and sequential harvesting (**Figure 1**). These strategies will need to be vineyard specific to match the topography, environmental conditions, cultivar, and style of wine desired, and it is likely that a changing combination will be required depending on the urgency of immediate threats versus addressing longer term sustainability. Additionally, grower, community and consumer values/pressures will need to be considered along with labour availability and other socio-economic factors. Most importantly, any adaptation measure should consider potential short- and long- term impacts on the environment. Sustainable productivity will require a balanced approach with environmental stewardship and the preservation of our resources for future generations (Doran, 2002).

### 2.1 Accessing cooler sites

Suitability maps based on bioclimatic indices have been developed for future climate scenarios. Of the 61 wine regions in Australia, it is expected that quality wine grape production will be affected in 21 regions by 2070 if appropriate adaptations are not implemented (Hall and Jones, 2009). The investment into cooler regions is already apparent, particularly in Tasmania where wine production is increasing by 10% each year.

Moving vineyards to cooler elevations is another likely scenario. Higher altitudes have lower night temperatures, potentially resulting in higher acidity and aroma in grape berries with lower alcohol in the wine (Van Leeuwen and Senguin, 2006). Delayed budbreak and bloom are also characteristic of the lower temperatures at increased elevations (Falcão et al., 2010). Vineyards that are currently located on sun-exposed slopes, may spread into sites that traditionally avoided because these sites were low in temperature. The particular fine-scale conditions including aspect, slope, wind exposure, soil drainage and proximity to water bodies will impact on suitability of a site, but often are not captured with macroclimatic modelling (Mosedale et al., 2016; Pipan et al., 2021). Regardless, shifts in location will entail high economic, environmental and social cost. Extension of vineyards into more marginal sites maybe at the expense of loss of natural habitats, impacting on biodiversity and natural water supplies.

### 2.2 Alternative cultivars, clones and rootstocks

Viticulture has the availability of hundreds of cultivars well suited to a wide range of climates, extending from cool, intermediate, warm to hot conditions (Jones, 2006). The conservation of current viticultural regions is possible by exploiting this diverse plant material. Finding the right cultivar or clone for a specific location is complex, however, and is dependent on the cultivar/clone’s climate niche and the distinctive wine style that is desired. Any one cultivar can produce quite different wine styles, with cooler climates resulting in lighter, fresher and crisper styles of higher acidity while warmer climates result in wines with more body, colour, higher alcohol and darker fruit characters. Growing a cultivar outside its climatic niche may decrease certainty in consistent productivity, quality as well as wine style. High quality Pinot Noir, for example, has a particularly narrow range of optimal average growing season temperature, at 14-16°C (Jones et al., 2005) and its future suitability to a particular location could be in doubt (Jones and Webb, 2010). A greater effort to match the climate to many of the lesser-known cultivars will greatly enhance the adoption of these cultivars in a changing climate. The allocation of specific rootstocks that are able to shift phenology and ripening rates (Van Leeuwen and Destrac-Irvine, 2017) will also be valuable but will require site specific research in line with cultivar credentials.

Drought tolerant cultivars may be considered for the future when water is scarce or too expensive. Likewise, late ripening cultivars may be required to address the expected advance of phenology (Duchêne et al., 2010). Choosing a cultivar to plant in a new vineyard today, knowing that it will experience a different climate in 20-30 years, is challenging and is also confounded with changing consumer preferences. Shiraz might be the iconic Australian wine for some time yet, but there is scope to bring in lesser-known cultivars or even newly bred cultivars that are better able to cope with climate change. To spread the risk, some winegrowers are adopting to plant several cultivars and/or rootstocks within a specific location.

Cultivars from countries such as Italy, are currently being planted in regions across Australia and their growth, productivity and wine production potential are being evaluated (Chalmers, 2019). In particular, the cultivar’s distinctive character is assessed along with consumer preference studies to better gauge its potential for premium wine production. The breeding of cultivars suited to hot climates from additional ongoing research has yielded interesting cultivars, producing wines with, for example, increased colour intensity that may be used on their own or for blending purposes (Dry et al., 2022). Specifically, disease resistant cultivars bred by CSIRO have been evaluated across both cool and warm growing regions with good performance. Second generation mildew-resistant cultivars is the current focus of the breeding programs and the microvine, a dwarf grapevine mutant that flowers continuously (Dry and Thomas, 2015), will speed up the process considerably.

Aside from climate, soil is an important component of the vine’s natural environment and the roots interact directly with the abiotic and biotic characteristics of the soil profile along a range of depths. Rootstocks offer resistance to phylloxera, nematodes and fungal pathogens but they also have varying tolerance to abiotic stresses (Marín et al., 2021). Vines with deeper and more extensive root systems are better able to access available underground water, while others have a greater intrinsic capacity to absorb water as a result of their higher fine root hydraulic conductivity and aquaporin activity (Gambetta et al., 2012). Rootstocks that are able to control vigour, or influence stomatal conductance through chemical or hydraulic signalling, can reduce vine transpiration and maintain plant water status. Drought tolerant rootstocks such as Ramsey, 110 Richter, the new generation M-series rootstocks, and salt-exclusion rootstocks such as 140 Ruggeri and 1103 Paulsen have been adopted to help vines cope with drought and soil salinity (Stevens and Walker, 2002; Pitt et al., 2018; Bianchi et al., 2020). Soil salinity has increased due to irrigation, of concern in the Murray River Valley of Australia, as high salinity impacts severely on growth and productivity. To improve tolerance to flooding, grafting onto Couderc 3309 resulted in less adverse impacts (Striegler et al., 1993). Rootstocks can also influence phenology and ripening (May, 1994; Walker et al., 2000). Breeding programs can target rootstocks that shift berry ripening into a lower temperature period enabling berry anthocyanin and acidity to be maintained. Trials have been implemented to assess rootstocks for their ability to reduce potassium uptake by vines and berries (Ollat et al., 2015; Walker and Clingeleffer, 2016; Xiao et al., 2020) considering that grape juice pH is correlated with juice potassium and high pH has negative consequences for flavour, wine stability and colour (Rogiers et al., 2017).

### 2.3 Irrigation

Increases in evaporative demand and declines in precipitation as a consequence of climate change result in greater irrigation requirements for the vineyard. However, in Australia, it is expected that the quantity and quality of water available for irrigation will be reduced (Murray-Darling Basin Authority, 2010). Drawing water from surface and groundwater sources has impacts on the surrounding natural environment as well as other agricultural industries and thus more judicious use of this precious resource is at the forefront of vineyard managers. The conversion from flood to drip irrigation in Australian grape growing regions has resulted in enormous water savings, and despite the additional energy required to run water pumps, the precision offered by drip irrigation has allowed the implementation of various mild deficit strategies to influence yield, berry composition and wine properties (Edwards and Clingeleffer, 2013). Regulated deficit irrigation (RDI) is another strategy used to limit competition between vegetative and reproductive growth in red cultivars and is usually applied between fruit set and veraison. While RDI and prolonged deficit reduced yield in Cabernet Sauvignon grown in a warm climate, there was no progressive reduction in yield over multiple seasons (Edwards and Clingeleffer, 2013). However, deficit irrigation in combination with elevated temperatures were detrimental to Shiraz wine phenolic substances and sensory traits (Bonada et al., 2015). Applying a water deficit prior to véraison may offset the delay in anthocyanin accumulation that can occur during high temperatures later in the season (Sadras and Moran, 2012). Investment in soil moisture sensors and methods to monitor vine water status will ensure that critical thresholds are not surpassed in managing the vineyard.

From the perspective of balancing canopy management and water-use efficiency, even though water savings are generally beneficial, if canopy development is impaired and bunch over-exposure occurs then it is suggested that sufficient water should be applied early in the season to allow full canopy development. The cooler microclimate because of the shading will lessen the severity of berry sunburn and shrivelling during late ripening. Hypoxia and cell death in the berries can also occur during the late ripening stage in some cultivars (Xiao et al., 2018b), and this is exacerbated by water stress (Bonada et al., 2013b; Bonada et al., 2015; Xiao et al., 2018a).

The loss of berry cell vitality was characterised using the cell vitality dye (Krasnow et al., 2008; Tilbrook and Tyerman, 2008; Fuentes et al., 2010), which was associated with the reduced electrical impedance of the berries (Caravia et al., 2015). The cell death inhibitor gene *VvBAP1* was associated with increased drought tolerance (Cao et al., 2019). Shiraz is a variety associated with severe hypoxia and cell death in berries (Xiao et al., 2018b), increased hydraulic resistance into the berry after véraison (Tilbrook and Tyerman, 2008), as well as greater propensity for backflow of water from the berry to the vine in the later stage of ripening (Tilbrook and Tyerman, 2009). Due to greater severity of hypoxia and a decline in respiration, the energy status of berries may be curtailed in the late ripening stages. These changes in berry energy status might be correlated with altered metabolism and the greater force to transport solutes into and within the berries. The hydraulic and energy status of the berries, and the vitality of berry cells, may provide more insight into berry ripening, potentially informing future irrigation and fertilisation strategies.

Practical safeguard measures to deal with drought include the use of polymers or physical covers to reduce evaporation from storage dams, prioritising high value blocks when water is limited. Monitoring water demand through soil or plant-based sensors in combination with weather data, will ensure sustainable water use and potentially water savings for heatwave events. Ensuring dripper spacing is frequent enough to prevent alternating wet and dry zones will allow consistent root growth to spread across a wide area.

#### 2.3.1 Irrigation during heatwaves

Irrigation is recommended prior to an impending heatwave to ensure that the optimal field capacity is maintained (Webb et al., 2010). Relative to the local climate and historical observations, a three-day period of unusual high maximum and minimum temperatures can be classified as a heatwave event (Nairn and Fawcett, 2015). During the heatwave, the vines should continue to be watered at regular intervals ensuring that transpiration is maintained to cool the canopy and bunches (Hayman et al., 2012). Application of water directly to the canopy is discussed below in 2.6 under hydro-cooling. The timing of the heatwave during the growing season will result in different consequences, with earlier heatwaves lowering fruit set and later heatwaves disrupting ripening. Heatwaves can cause vines to ‘shut down’; that is, photosynthesis stops, due to stomatal closure, so that water can be conserved, and thus the rate of ripening slows (Greer and Weedon, 2013). Depending on the timing, severity and duration of the heatwave, it may take several weeks for the vine to resume its normal photosynthetic activity resulting in overall low-quality fruit. In contrast, it has been suggested that dry soils brought about by higher temperatures and drought may advance grape maturity (Webb et al., 2012) associated with ABA production by the roots (Davies et al., 2000), a hormone correlated with ripening (Wheeler et al., 2009). Moreover, dry soils fluctuate in temperature to a greater extent during the day hence vine roots may experience warmer temperatures (Rogiers et al., 2014) when the soils are dry.

#### 2.3.2 Post-harvest irrigation

Post-harvest irrigation has the benefit of prolonging leaf photosynthesis if conditions are warm enough so that vine carbohydrates can be replenished (Loescher et al., 1990). Grapevines rely on the stored reserves during early season root, shoot and inflorescence growth and a long post-harvest period with adequate soil moisture allows carbon capture through photosynthesis and may even encourage a new flush of fine root growth to aid in the uptake of nutrients (Mahmud, 2016). The timing of leaf senescence to some extent determines the length of carbohydrate reserve accumulation during the post-harvest period and this can be considerable in warm-climate viticulture.

#### 2.3.3 Dry winters

Rainfall has been declining in late autumn and early winter in south-eastern Australia (Cai and Cowan, 2013) and may have negative impacts during budburst and spring canopy development. A recent study investigated the effects of low winter rainfall on vine growth and wine quality using rainout shelters (Bonada et al., 2017). Vines were irrigated with micro-sprinklers or drippers at different timepoints during the winter and it was evident that waiting to refill the profile until budburst resulted in excessive vegetative growth with negative consequences on yield and wine composition. It is likely that root growth dynamics and root longevity is altered by the changes in precipitation and irrigation patterns and this will have consequences on overall vine performance. Bonada et al. (2017) recommended that the soil profile be maintained throughout winter rather than delaying until spring to irrigate and refill the soil profile.

### 2.4 Delayed pruning

Delayed maturation of the berries into the cooler period of the growing season and decompressing the harvest window can be encouraged by delayed pruning. Applying pruning after budburst can delay véraison, allowing ripening to occur during cooler months. Pruning carried out at the 10 cm shoot length stage, following mechanical winter spur pruning, achieved a reduction in sugar accumulation in Sangiovese berries (Palliotti et al., 2017). Shiraz berries from vines pruned at the 2-3 leaf stage reached sugar ripeness up to two weeks later than those with winter pruning applied and attained an improved anthocyanin to sugar ratio (Moran et al., 2017). This strategy was favourable for balancing tannin accumulation, colour intensity, fruit aroma and flavours (Moran et al., 2018).

In addition, delayed pruning can minimize the risk of spring frost damage of the young spring shoots (Friend and Trought, 2007) and improve pollination and fertilisation, by inducing flowering to occur in warmer months. Delayed spur-pruning of the previous season’s apical nodes inhibits the development of basal nodes through apical dominance. Pruning carried out three months later than the usual winter pruning time increased yield by 60 to 90%, in Merlot, over a three-season trial (Petrie et al., 2017). However, the impact of delayed pruning on yield was considered to be dependent on interrelated factors such as cultivar, seasonal conditions as well as timing and severity of the pruning applied. Hence further development and assessment of this strategy is needed.

### 2.5 Row orientation, training systems and canopy manipulation

#### 2.5.1 Vine balance

Balancing yield with new biomass growth is critical to maintaining long-term sustainable productivity. Nutrient and carbohydrate reserves are essential for supporting early spring growth prior to flowering and are subsequently refilled within the vine prior to ripening (Rogiers et al., 2011b). Heat and drought can influence the quantity of the photoassimilate that is fixed and then distributed to the three growing sinks (roots, shoots and bunches), as well as the reserve pool, predominantly sequestered as starch in the vine structural root system (Rogiers et al., 2011a).

Vine balance can be achieved by pruning of the dormant vines and thinning of the growing leaves/shoots. The number of nodes to be retained during winter pruning can affect vigour, yield, fruit composition and long-term productivity of the vines. Factors such as cultivar, climate and other management influences should be considered when determining the number of nodes retained. Keeping long-term records on yield and pruning weight will aid in monitoring vine performance for reliable vine balance assessment and crop load calculation. Leaf removal and shoot thinning can influence ripening of the fruit to varying degrees, depending on the cultivar, growing conditions, timing and severity of the thinning applied (De Bei et al., 2019). Excessive leaf removal around the bunch zone may result in sunburn of berries. Likewise, shoot thinning of Semillon at the 8-9 leaf stage in a hot Australian climate did not alter berry composition, probably because vine balance was not altered (De Bei et al., 2020). Further research on thinning techniques is required to assess the sustainable effects of this strategy.

#### 2.5.2 Natural shading

The geometry of the canopy can be manipulated to ensure that the microclimate of the bunch zone is well-suited to the development of desirable berry attributes. Adequate solar radiation is required for optimal photosynthesis, bud fertility, and berry development, including polyphenol accumulation (Berli et al., 2008). However, over-exposure of leaves may result in photo-inhibition of photosynthesis (Greer et al., 1986) and leaf necrosis and mortality. Sunburnt fruit leads to increased bitterness with browning of the wine, and damaged skin can lead to pathogen invasion. Dark cultivars can experience berry temperature 15°C higher than ambient temperature and thus alterations in the primary and secondary metabolites important to wine quality are likely (Bergqvist et al., 2001). The excessive exposure of leaves and fruit to direct incoming and reflected radiation can be addressed by row orientation, vine architecture, planting density and canopy management. The hot afternoon sun can be avoided by orientating rows in the east-west direction as opposed to the north-south direction (Buesa et al., 2020). Allowing canopies to sprawl so that fruit are shaded will minimise sunburn and shrivelling in hot, sunny climates. While the natural shading may decrease air circulation and increase disease pressure, this may not be an issue in dry regions. Because light is important to anthocyanin production, shading may decrease colour in the red cultivars hence the balance between temperature and light will need to be considered. Removal of the leaves above the bunchzone of cvs. Bobal and Tempranillo vines prior to veraison, and grown under mild water stress, limited the accumulation of anthocyanins more than total soluble solids and was thus not recommended as a method to delay harvest (Buesa et al., 2018). Similarly, avoiding shoot trimming or leaf removal will decrease sun damage and also save on labour costs. Vertical Shoot Positioning (VSP) is not recommended in hot, dry conditions and when sun damage is an issue. Foliage wires can, however, be used to minimise canopy displacement caused by wind.

Pergola type systems that allow the spread of leaves above the hanging fruit are ideal for providing shade, and ventilation simultaneously. In contrast, the Gobelet, also known as the bush vine, is an ancient architectural system still used in the dry regions of Spain with reduced leaf area (Van Leeuwen et al., 2019). It, however, has the drawback of low productivity and is not conducive to mechanical harvesting. The bush vine tends to have a short trunk, however, trunk height can be manipulated to raise bunches away from the soil surface where temperatures may be warmer (Van Leeuwen et al., 2019)

### 2.6 Shade structures and hydro-cooling

The interactive effect of temperature and photon flux densities on reproductive development in Shiraz vines was assessed in response to several densities of shade cloth over three seasons (Abeysinghe et al., 2018). During severe heat events the 50% shade treatments were able to reduce canopy temperatures significantly, by approximately 4-5°C. While berry growth was delayed by a 50% shade cover, maximum berry size and the rate of sugar accumulation was not affected. It was interesting to note that shade cover up to 50% had no impact on the ripening process when there were no heat events. The berries had lower sugar concentrations due to greater water content and cell vitality was maintained, but most notably the wines from the fruit resulted in lower alcohol without reduced anthocyanin concentrations. Alongside density, the net’s colour has an important impact on the spectral wavelengths that the canopy and fruit are exposed to, thus impacting on vegetative growth and reproductive development, including fruit composition (González et al., 2015).

Overhead sprinklers and misting may reduce heat and water stress as they are able to cool both canopies and berries. Semillon is a cultivar that is particularly sensitive to heat stress both during flowering and ripening, with reduced photosynthesis due to both stomatal and nonstomatal limitations and requiring up to 2 weeks to recover (Greer and Weston, 2010; Greer and Weedon, 2013). Hydrocooling, activated at a threshold temperature of 35°C, extended the period for leaf and berry expansion so that berries were larger. Canopy temperatures were lower, net CO_2_ assimilation was higher and berry TSS was also slightly elevated (Greer and Weedon, 2014)

The cost and availability of water for hydrocooling may be an issue and adequate planning so that enough water is available for the entire season is required. The increase in disease pressure is likely to be low in heatwave conditions, but if conditions are warm and humid for a protracted period this may become an issue. The water quality should be adequate as saline water will result in leaf burn and defoliation.

### 2.7 Antitranspirants, sunscreens and film-forming barriers

Plant tissue films have been available to viticulture for several decades and have been trialled for their capacity to maintain tissue hydration (e.g. di-1-*p*-menthene: Palliotti et al., 2013; Gatti et al., 2016; Rogiers and Fahey, 2019), prevent sunburn (e.g. kaolin: Dinis et al., 2016; Conde et al., 2016; Conde et al., 2018), decrease disease incidence (e.g. chitosan: Meng et al., 2008) or even to ameliorate smoke taint (e.g. kaolin: Van der Hulst et al., 2019). These films are derived from natural or synthetic origins and, depending on their composition, time of application and concentration, may or may not be beneficial. When compared to protective netting, these particle film-forming and antitranspirant products may reduce fruit quality and consumer acceptance (Brillante et al., 2016). In a smoke taint study, several materials were tested for their ability to prevent absorption of gaseous phenols but it was noted that they did not provide much protection (Culbert et al., 2021).

### 2.8 Sustainable vineyard floor management

#### 2.8.1 Inter-row and under-row vegetation

Bare soil as a result of cultivation contributes significantly to vineyard heating, water and carbon loss, soil erosion and greenhouse gas emissions (Santos et al., 2020). Allowing the resident vegetation to grow or planting cover crops can improve water infiltration, soil organic matter and microbial function (Celette et al., 2008; Steenwerth and Belina, 2008). Inter-row and under-row vegetation can encourage soil biodiversity and lessen disease pressure (Garcia et al., 2018). To minimise the competition for water between the vines and the inter-row plants they can be slashed or allowed to dry (Webb et al., 2010). Moreover, planting native species alongside a vineyard has the benefits of reducing wind, salinity, erosion and providing ecosystem services (Viers et al., 2013).

In regions with adequate rainfall, legume cover crops can supply soil organic matter and biologically fixed N to the grapevine (Ball et al., 2020). Nitrogen application to soil in the form of synthetic fertilisers, manures, composts or mulches can lead to nitrous oxide emissions, a particularly potent greenhouse gas (Longbottom and Petrie, 2015). Targeting the application of N during the phase of active nutrient uptake (before flowering and after harvest when there is a long post-harvest period) will reduce excessive N in the soil and curb nitrous oxide emissions. Employing drip irrigation instead of furrow or flood irrigation will also curb emissions as microbial activity leading to denitrification is reduced (Suddick et al., 2011).

#### 2.8.2 Mulches, composts and soil conditioners

**Figure 2:**
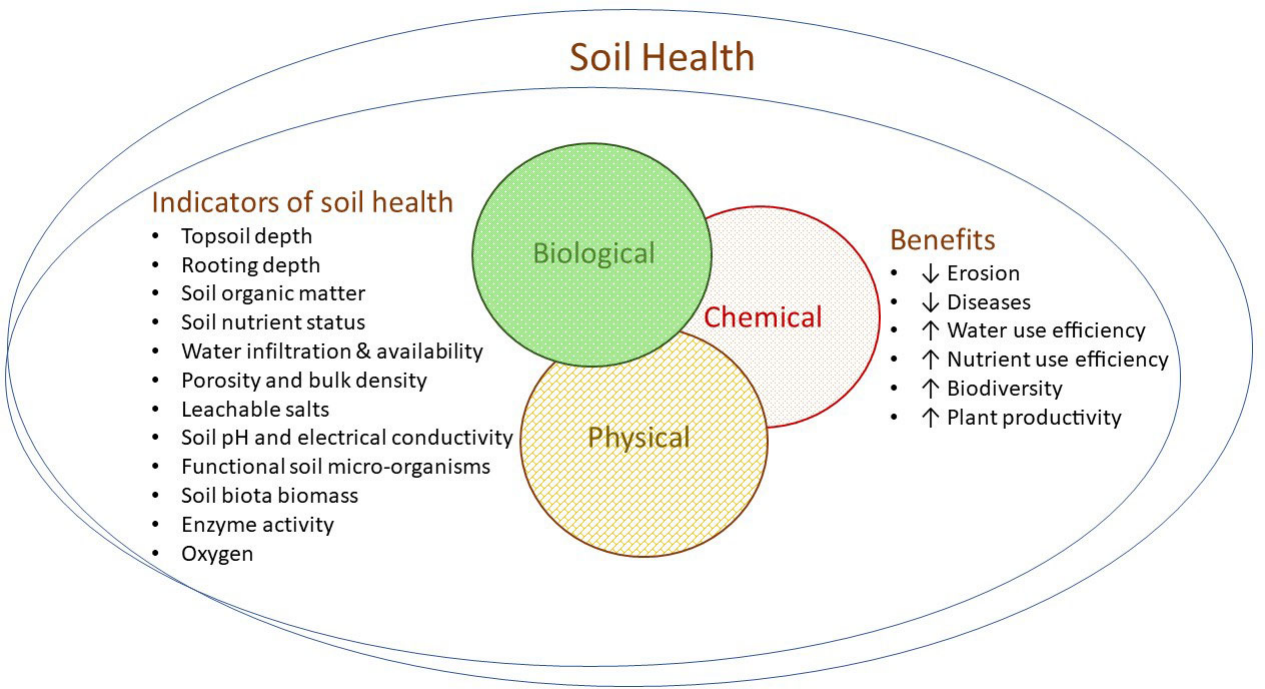
Indicators and benefits of optimum soil health. Soil health is comprised of the interactive physical, chemical and biological spheres and can be assessed through various measurable indicators and resulting benefits in the vineyard.

Undervine mulch, such as straw or vineyard prunings, has many benefits, aside from weed control, including the amelioration of soil water evaporation (López-Urrea et al., 2020), the regulation of soil temperature and improving biodiversity. Improving the carbon content of the soil by adding mulch and composts not only offsets carbon emissions but also improves soil structure and the water holding capacity of the soil. Likewise, composts increase nutrient reserves, the cation exchange capacity, and reduce the requirement for synthetic fertilisers. Organic matter supports soil biodiversity, which in turn suppresses pathogens and promotes beneficial as opposed to parasitic organisms. **Figure 2** provides an overview of factors driving soil health and indicators for soil health assessment. Microbes are especially beneficial at the recycling of nutrients from organic matter and minerals. Mulches applied on top of the manure or compost further reduces the release of nutrients such as nitrogen to the atmosphere and also helps control root-zone temperature. The characteristics of the soil amendment including its pH, EC, nutrient and heavy metal content should be considered prior to application. Composted grape marc, for instance, contains potassium and this has the potential to increase the pH of the grape must so that acid additions are required in the winery (Rogiers et al., 2017). That said, the addition of grape marc to soil in New Zealand had minor effects on juice K and pH (Mundy and Agnew, 2002).

Organic amendments such as biochar and brown coal waste (BCW) are a source of soil organic matter, which can influence the soil microclimate, microbial community structure, biomass turnover and mineralisation of nutrients (Amoah-Antwi et al., 2020). Coal derived humate is potentially effective as a soil conditioner in improving aggregate stability of acidic and sodic soils against adverse effects of cyclic seasonal wetting and drying conditions (Imbufe et al., 2005). Biochar is a carbon-rich material produced by heating organic material through the pyrolysis process in an oxygen limited environment. Feedstock and pyrolysis conditions (temperature, heating rate, oxygen supply, pressure, residence time, cooling down procedure) can result in biochars with differing chemical and physical properties (Fryda and Visser, 2015). Therefore, they are heterogeneous materials with a diverse range of properties that change over time. Biochar may improve soil function, increase nutrient availability to plants, promote plant productivity, remediate organic/inorganic contaminants, reduce N_2_O emissions, increase soil pH and lead to the net removal of carbon from the atmosphere (Laird, 2008; Kolton et al., 2017). Biochar’s macropores filled with adsorbed nutrients create a habitat for beneficial microorganisms (Atkinson et al., 2010). Biochar can provide protection against both root and foliar plant diseases through the direct manipulation of bacterial communities or by induced plant resilience (Elad et al., 2011). In vineyards, biochar may increase soil fertility, productivity or both (Baronti et al., 2014; Schmidt et al., 2014; Genesio et al., 2015; Mackie et al., 2015; Giagnoni et al., 2019; García-Jaramillo et al., 2021). Notably, a long-term study over ten years showed evidence of increases in soil water content and plant water status (Baronti et al., 2022). However further research is required using different parent materials and pyrolysis conditions to gain a better understanding of the potential for improving plant resilience and maintaining fruit quality under climate change.

### 2.9 Biostimulants

Application of biostimulants to grapevines have a high potential for promoting nutrient uptake, tolerance to biotic and abiotic stress and improving fruit yield and quality (Monteiro et al., 2022). Biostimulants are defined as “any substance or microorganism applied to plants with the aim to enhance nutrition efficiency, abiotic stress tolerance and/or crop quality traits, regardless of its nutrients content” (Du Jardin, 2015). Biostimulants commonly used in vineyards include seaweed extracts, humic substances, chitosan, exudates, and other plant extracts (Cataldo et al., 2022). Seaweed extracts contain macro- and micro-nutrients, amino acids, vitamins, auxins, abscisic acid and cytokinins. Likewise, brown sea weeds (*Ascophyllum nodosum* L.) have been shown to improve tolerance against biotic and abiotic stresses (Salvi et al., 2019; Taskos et al., 2019).

### 2.10 Sequential harvesting

Wine alcohol can be reduced through the simple method of adding water to the juice and amendments to the Australia New Zealand Food Standards Code now allows limited addition of water to high sugar must above 24.3°Brix. Alternatively, alcohol can be removed from wine using vacuum distillation or membrane systems based on reverse osmosis and evaporative perstraction, however the methods are not eco-sustainable and these wines tend to lack aroma through the loss of desirable volatile compounds responsible for fruity and floral characters (Longo et al., 2018). Other strategies include the use of yeast strains tailored for lower ethanol yields (Ristic et al., 2016; Hranilovic et al., 2020). Grapes harvested at a lower sugar level may have insufficient phenolic and aroma attributes that are characteristic of more mature fruit, and they may have too much acid and an undesirable herbaceous character. This can potentially be overcome by blending a low and high alcoholic wine so that acid levels are balanced and ripe flavours mask the unripe attributes. Following two sequential harvests, blending wines made from less ripe grapes with wine vinified from riper fruit resulted in a wine with similar sensory profiles of the later harvested fruit but with a lower alcohol content (Longo et al., 2018). This particular investigation was conducted on Petit Verdot and Verdelho, but the positive outcome certainly warrants further studies on other cultivars.

### 2.11 Technology, sensors, AI and online tools

Information on vineyard status can be obtained through sensors placed in contact with the soil or plant. For instance, soil nutrients can be assessed using ion-selective electrodes and ion-sensitive field-effect transistors (Adamchuck and Rossel, 2010). Direct measurement of plant water status for irrigation scheduling is more accurate than soil moisture monitoring alone as sensor placement may not be representative of full field conditions; moreover, plant measurement integrates evaporative demand. The most accurate method for assessing plant water potential is the pressure chamber, however this method is not conducive to automation. Dendrometers (Clonch et al., 2021), sap flow (Mancha et al., 2021), and acoustic sensors (Oletic et al., 2020) provide information on plant water status, and if temperature sensitivity, set-up inaccuracies and data interpretation difficulties can be overcome, these tools may be useful when correctly calibrated. Additionally, climate sensors alongside leaf temperature and wetness sensors provide continuous data on pest and disease pressure. These data can be captured using wireless sensor network (WSN) technologies that allow the real-time remote monitoring of several areas within the vineyard.

While contact sensors have their place in vineyard management, contactless sensors are the new focus of the viticulture industry. Satellite imagery allows monitoring at a large scale however is limited by satellite orbit coverage patterns, visiting times, clouds and spectral resolution. Remote sensing using unmanned aerial vehicles (UAVs) or ground vehicles equipped with GPS and an array of sensors is capable of the collection of high-resolution data (mm) on phenology, water status, vigour, pest and disease incidence, weeds, yield and maturity (Seng et al., 2018; Maes and Steppe, 2019; Sassu et al., 2021). Thermal (Far-IR), RGB (red, green, blue), multispectral, hyperspectral sensors and chlorophyll fluorescence systems have all been applied to assess plant status and reproductive parameters (Matese et al., 2015; Tardaguila et al., 2021). Light detection and ranging (LiDAR) is appropriate for the determination of leaf area index and can be used to assess dieback as a result of trunk diseases (Ouyang et al., 2021). Hyperspectral and near-infrared (NIR) spectroscopy have been useful for the assessment of real-time berry composition during ripening (González-Caballero et al., 2012; Power et al., 2019). Additionally, both thermal imaging and NIR spectroscopy have been developed for real-time plant water status assessment (De Bei et al., 2011; Diago et al., 2018), with automated variable drip irrigation to address low-performing areas within a vineyard on the radar. Thus, high-resolution monitoring allows for management that takes into consideration the natural variability in the block (Fuentes and Gago, 2022).

One of the greatest challenges that remains is the processing and integration of the large data sets acquired by these monitoring technologies (Manfreda et al., 2018), allowing a targeted management plan to be effected. AI and machine learning (ML) models are able to overcome these limitations and have been rapidly advancing in viticulture (Seng et al., 2018; Fuentes and Gago, 2022). These approaches can result in intelligent recommendations for efficient water, pesticide and nutrient use not only to target yield and composition but also to deal with temporal and spatial variability. Moreover, robotic innovations are currently under development for the monitoring, pruning, spraying and weeding of vineyards (Matese et al., 2015). Thus, these technologies allow for targeted treatment with improvements in efficiency and reductions in environmental repercussions.

**Figure 3:**
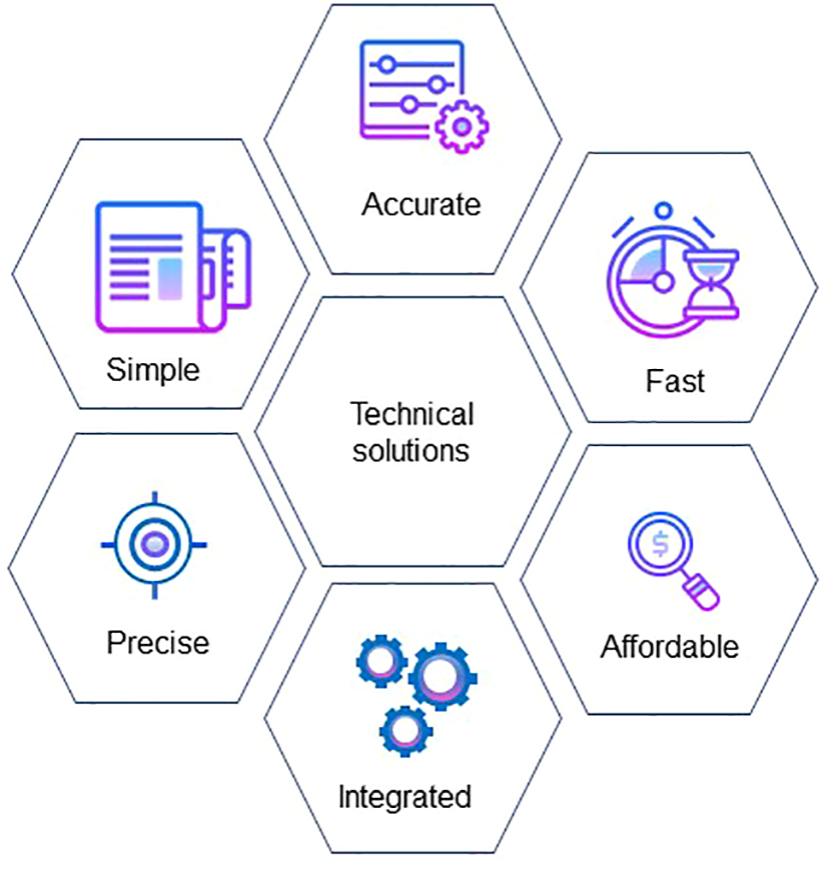
Minimum requirements for maximum adoption of technical solutions by the viticulture industry.

The widespread use of smartphones and apps allow growers rapid and cost-effective assessments of individual vines within the vineyard. A smartphone with an attached thermal camera showed promise for assessing vine water status and to aid in irrigation scheduling (Petrie et al., 2019). Canopy vigour assessment through the VitiCanopy^®^ app has GPS capabilities making it appropriate to map spatial canopy architecture (De Bei et al., 2016), and early prediction of yield is possible at flowering (Aquino et al., 2015) and berry (Liu et al., 2020) quantification. ML has also been applied for the detection of smoke contamination in berries using NIR spectroscopy (Fuentes and Tongson, 2018; Summerson et al., 2021). Furthermore, computer vision and deep learning were applied for the detection of nutrient disorders in grapevines (Debnath et al., 2021). RGB images of nutrient deficiencies were taken as symptoms progressed and these were used to develop, train and test models for the incorporation onto a smartphone. Flexible and rapid adjustment to the challenges posed by variable weather patterns can now be addressed through timely online notification systems, including SMS messages to a phone. There has been significant investment in the forecasting of heatwaves and communication to growers so that they are able to prepare for such events. For maximum adoption, technical solutions need to be simple, rapid, affordable, accurate, precise and integrated with other data gathering platforms (**Figure 3**).

## 3 Summary

**Figure 4:**
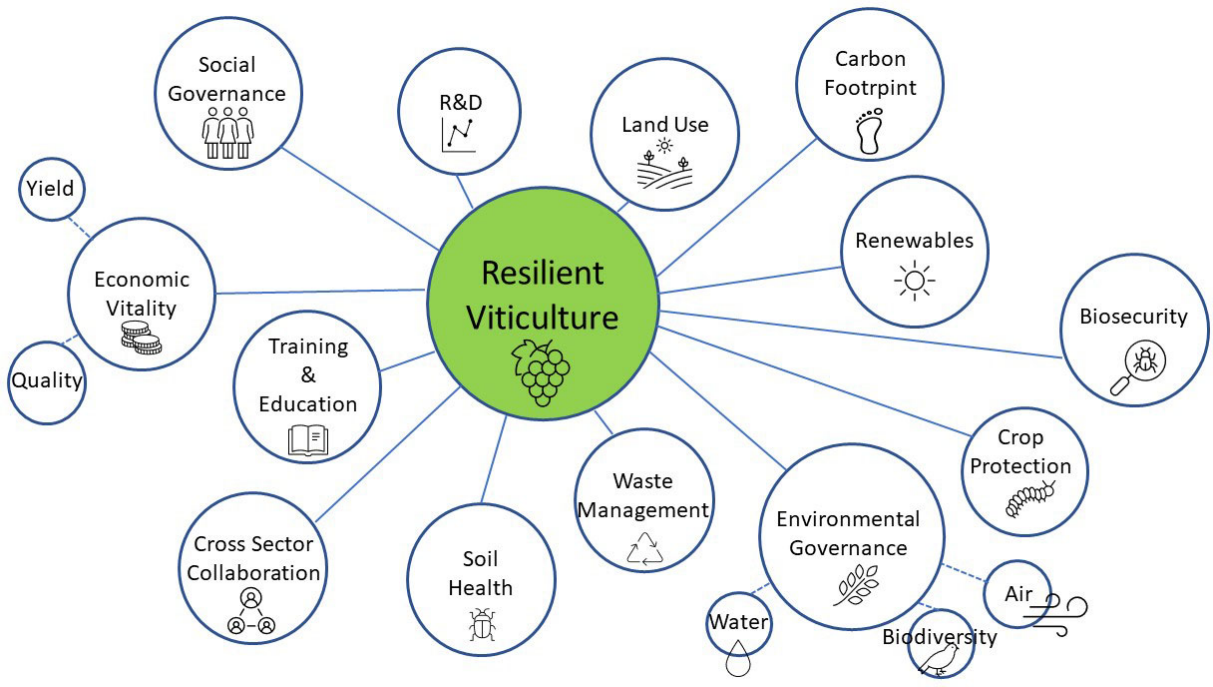
Resilient viticulture is reliant on a number of environmental, economic and social factors.

The Australian wine industry is already implementing site-specific adaptive measures to deal with unpredictable weather, droughts and rising temperatures. Growers are conscious of their environmental footprint, their long-term sustainability, and are balancing these visions with economic viability (**Figure 4**). The increasing awareness of sustainable management practises will result in overall improvements in environmental equilibrium and soil health for future generations. As they become more economical and user-friendly, technical innovations will be readily adopted and integrated with more traditional approaches to vineyard management. However, long-term field trials are required to fine-tune these adaptive strategies to particular situations. Additionally, the inclusion of stakeholders in a co-design framework for future R&D will accelerate the adoption of these mitigation strategies.

## Author contributions

Author contributions are as follows: Conceptualisation, investigation, visualisation, writing- original draft preparation: SR; Review and editing DG, ZX, YL, and TB. All authors have read and agreed to the published version of the manuscript.
